# Premature Adrenarche in Children with Prader-Willi Syndrome Treated with Recombinant Human Growth Hormone Seems to Not Influence the Course of Central Puberty and the Efficacy and Safety of the Therapy

**DOI:** 10.3390/life10100237

**Published:** 2020-10-10

**Authors:** Agnieszka Lecka-Ambroziak, Marta Wysocka-Mincewicz, Kamila Marszałek-Dziuba, Agnieszka Rudzka-Kocjan, Mieczysław Szalecki

**Affiliations:** 1 Department of Endocrinology and Diabetology, The Children’s Memorial Health Institute, 04-730 Warsaw, Poland; m.wysocka@ipczd.pl (M.W.-M.); oddzial.endokrynologia@ipczd.pl (K.M.-D.); a.rudzka-kocjan@ipczd.pl (A.R.-K.); m.szalecki@ipczd.pl (M.S.); 2 Collegium Medicum, Jan Kochanowski University (JKU), 25-369 Kielce, Poland

**Keywords:** Prader-Willi syndrome, hypogonadotrophic hypogonadism, premature adrenarche, central precocious puberty, recombinant human growth hormone treatment

## Abstract

Puberty in children with Prader-Willi syndrome (PWS) is usually delayed and/or incomplete but in some patients premature/early adrenarche is observed. We assessed the premature adrenarche (PA) in PWS patients during the recombinant human growth hormone (rhGH) therapy and influence of PA on the course of central puberty (CP), rhGH efficacy and safety, and patients’ metabolic state. Forty-nine PWS patients were treated with rhGH, 11 presented with PA (group 1) and 14 had normal course of adrenarche (group 2). PA was observed in 22.5% of the PWS children treated with rhGH. The mean time between the rhGH start and the adrenarche, the rhGH dose, the growth velocity and the insulin-like growth factor 1 SD (IGF1 SD) during the treatment, as well as the time of CP, final height SD and BMI SD were similar in both groups. There were also no significant differences in the metabolic assessment—the oral glucose tolerance test (OGTT) and lipid profile results. PA may be a part of the clinical picture of PWS, apart from hypogonadotrophic hypogonadism and it seems to have no influence on CP in PWS patients. The rhGH efficacy and safety were comparable in the patients with PA and the normal course of adrenarche.

## 1. Introduction

Prader-Willi syndrome (PWS) is a first recognized human genetic imprinting disorder with the prevalence of 1 in 15,000 to 1 in 30,000. The genetic mechanism is lack of paternally-inherited genes on chromosome 15q11-q13, that results from paternal deletion (DEL 15, 70%), maternal uniparental disomy (UPD 15, 15–30%) or imprinting center defect (IC, estimated for 1%) [1,2,3].

The main clinical features of PWS are, besides dysmorphic features, hypotonia, psychomotor delay, difficulties in feeding during the first year of life with failure to thrive, followed by lack of satiety and obesity developing from the early childhood, hypogonadotrophic hypogonadism, with almost universal cryptorchidism in males, short stature, cognitive and behavior dysfunction. The clinical symptoms appear to derive from hypothalamic function disruption. The rhGH treatment together with a multidisciplinary care has been a well-established approach for PWS patients [4,5,6,7,8,9].

Recent studies have shown more complicated mechanisms of puberty disorders in PWS. Hypogonadism in PWS seems to be actually of both central and primary origin, with differences also between male and female groups. Interestingly, fertility can be sustained in a few PWS female patients [10,11,12,13,14,15,16]. Moreover there have been a few cases of central precocious puberty (CPP) in both sexes reported. It can be linked to loss of *MKRN3* (makorin RING-finger protein 3) gene, within the 15q11-q13 region, which has been found to be one of the most commonly recognized genetic reason for CPP in the general population [17,18,19,20]. It has also been reported that premature adrenarche (PA) can be present in a significant part of PWS patients [21,22,23].

The main aim of the work was the assessment of frequency of PA in PWS patients during the recombinant human growth hormone (rhGH) therapy as well as influence of PA on the timing and course of central puberty (CP), the rhGH treatment efficacy and safety, and patients’ metabolic state.

## 2. Material and Methods

Forty-nine PWS patients were treated with rhGH, 11 (22.5%) presented with PA (group 1) and 14 had normal course of adrenarche (group 2). The mean age of the patients at the end of the study was 12.1 ± 6.8 years, 14 were still in the prepubertal age with no symptoms of adrenarche or CP at the end of the study.

The data on the adrenarche, CP, the rhGH treatment and metabolic assessment were analyzed retrospectively.

The puberty stages were assessed according to the Tanner scale. The beginning of adrenarche is clinically defined as the start of pubarche or axillarche, that may be assisted by additional features, such as acne, intense body odor and increase of growth velocity. The beginning of CP is defined as the start of thelarche development in girls, and testes enlargment >3 mL in boys. Both adrenarche and CP should start above the age of 8 years in girls and 9 years in boys.

The main first symptom of PA in our patients was premature pubarche (PP).

The height and body mass index (BMI) were assessed according to the Polish growth and BMI standards charts, the bone age was assessed with the Greulich–Pyle method. The insulin-like growth factor 1 (IGF1), androgens, gonadotrophins (luteinizing and follicle stimulating hormones, LH and FSH) and insulin levels were evaluated with a radioimmunoassay technique. The oral glucose tolerance test (OGTT) was performed with the glucose dose of 1.75 g/kg (maximal dose 75 g). The dose of subcutaneous gonadotrophin-releasing hormone (GnRH) for the stimulation test was 75 μg/m^2^ (maximal dose 100 μg).

The study was approved by the CMHI Bioethics Committee, 7/KBE/2019, 20 March 2019.

## 3. Data Analysis

Statistical analyses were performed using statistical software Statistica 6.0 Statsoft Company. Results are expressed as mean values and standard deviation scores (±SDS). Data were checked for normality of distribution using Shapiro–Wilcs test, data with skewness were log or square transformed to normal distribution if possible. Differences between the two groups were tested by unpaired *t*-Student test or Mann–Whitney U test, as appropriate. Analysis of data before and during rhGH-treatment was done using paired samples *t*-Student test or Wilcoxon rank test, as appropriate. A *p* level < 0.05 was recognized as statistically significant.

## 4. Results

Group 1 n = 11, mean age of PWS diagnosis 1.4 ± 1.1 years, most of the patients were diagnosed with DEL 15 (n = 8). 7 patients had intrauterine growth retardation (IUGR). Cryptorchidism was present in 2 out of 3 boys, orchidopexy was performed in one boy, aged 5 years. The clinical characteristics of group 1 are presented in Table 1.

The beginning of adrenarche, PP, was observed at the mean age of 6.9 ± 0.7 years, in 2 girls before the commencement of the rhGH treatment.

The mean serum androgens’ levels were: dehydroepiandrosterone-sulphate (DHEAS) 1228.4 ± 531.1 ng/mL, androstenedione 84.5 ± 48.4 ng/dL, testosterone 102.6 ± 79.9 pg/mL. The urine steroid profile, performed in 5 patients, showed slightly elevated androgens’ metabolites in 3, definitely elevated androgens’ metabolites only in one girl, who developed central precocious puberty (CPP). Bone age/chronological age ratio (BA/CA) was 1.0 ± 0.2 at the CA of 7.0 ± 0.8 years. The above data are summarized in Table 2.

The metabolic assessment was performed in 10 patients after the mean time of therapy of 5.3 ± 2.5 years, at the mean age of 10.1 ± 4.8 years, the data are presented in Table 3.

The course of adrenarche in the analyzed patients had not been rapid, the first symptoms of CP occurred at the mean age of 12.2 ± 2.2 years in 8 patients. CP was delayed in 4 patients. The basal gonadotrophins’ levels were evaluated in 9 patients aged 12.6 ± 2.2 years and showed LH and FSH levels of 1.4 ± 1.0 and 3.3 ± 2.3 IU/mL. The GnRH test performed in 6 patients, aged 7.4–16.2 years, revealed the peak LH and FSH of 8.8 ± 7.5 and 10.4 ± 3.7. The GnRH test was done after the mean time of 5.5 ± 2.3 years after adrenarche start and 0.4 ± 1.4 years regarding CP start.

In two girls, the treatment with estrogen and progestogen was introduced at the age of 18 and 16.4 years. During the rhGH therapy, scoliosis was observed in 6 patients (55%), one boy had transient central hypothyroidism. Two girls had severe behavioral disorders and needed pharmacological treatment.

### Case Report of PA Followed by CPP

One girl (patient number 10), with genetic diagnosis of changed methylation pattern of *SNRPN*, presented with PA (PP) at the age of 6.8 years, followed by CPP with thelarche development at the age of 7.1 years. She was treated with a relatively high rhGH dose—0.86 IU/kg/week (0.041 mg/kg/day), with a good response, but higher IGF1 levels (3.14 and 4.43 SDS). The metabolic assessment at the age of the CPP showed an elevated insulin fasting level in the OGTT, HOMA-IR 3.1 and a normal lipid profile. The patient was closely followed-up and did not show a rapid progression of CPP. The genetic analysis was broad at the time, with no *MKRN3* gene mutations found.

Group 2 n = 14, mean age of PWS diagnosis 3.7 ± 2.5 years: DEL 15 in 9 patients, UPD 15 in 1 patient and abnormality in methylation pattern of *SNRPN* in 4 patients. Three patients were born prematurely (32–36 weeks of pregnancy), half of the group had IUGR. Cryptorchidism was presented in 7 out of 9 boys, all 7 boys underwent orchidopexy at the age 0.9–15 years.

The beginning of adrenarche in one girl started within the period without the rhGH therapy (due to obesity) and in one boy before the rhGH treatment.

The BA/CA ratio at the age of 12.2 ± 1.7 was 1.0 ± 0.1. The OGTT and lipid profile assessments were performed at the age of 13.9 ± 3.2 years, after 6.5 ± 2.0 years of the rhGH therapy. The OGTT showed impaired fasting glucose in 1 case, impaired glucose tolerance in 1 case, insulin resistance in 5 patients, the mean HOMA-IR was 3.0 ± 2.0.

5 patients had elevated levels of total cholesterol with elevated LDL in 2; in one patient there was decreased level of high density lipoprotein cholesterol (HDL).

The sex steroid supplementation was introduced in 6 patients (4 girls and 2 boys) at the age of 12.0–19.3 years due to delayed puberty start or lack of puberty progression. Scoliosis was observed in 8 patients (57%), one boy presented transient central hypothyroidism.

Six patients (1 girl) had severe behavioral disorders that were treated pharmacologically.

## 5. Comparison Group 1 vs. Group 2

Adrenarche started significantly earlier in the group with PA in comparison to the group with normal adrenarche. The group of children with PA were younger at the time of the diagnosis and, as expected, started the rhGH therapy earlier. The age difference could have also led to the difference in BMI SDS and IGF1 SDS before the treatment, the mean BMI SDS was lower (*p* = 0.01) and IGF1 SDS (*p* = 0.04) was higher in the first group. This is consistent with the PWS clinical features, as both growth deficiency and increased appetite, leading to obesity in PWS, seem to increase with age. However, there was no statistical difference in the height SDS before the therapy, in spite of the later rhGH start in group 2. Interestingly, there was no difference in the time of the rhGH therapy and the commencement of adrenarche (slightly above 3.5 years: 3.8 vs. 3.7 years). The rhGH dose was also similar in the both groups and we have not found any statistical differences in the height SDS, BMI SDS, GV and IGF1 SDS during the therapy. The BA/CA ratio was close to 1.0 in both groups.

CP occurred at a similar age in 73% of the PA group and 57% of the group with normal adrenarche, regardless of the age of adrenarche, except one girl with CPP. The sex steroids treatment, due to delayed or incomplete puberty, was implemented in 2 girls in the group 1 and in 6 patients in the group 2. It can be explained by the younger age of patients in the PA group. The above data are presented in Table 4 and Table 5.

There was no difference in the height SDS at the last visit, as well as in the final height SDS achieved by 6 patients from group 1 and 12 patients from group 2 (−1.8 vs. −1.2, *p* = 0.40). Although the last BMI SDS value was higher in group 2, the difference didn’t reach the statistical significance, *p* = 0.12. The data regarding the last visit’s parameters are presented in Table 6.

According to the metabolic assessment in the 2 groups of patients we have found no differences in the OGTT, HOMA-IR, lipid profile values, performed at the mean age of 10.1 and 13.9, after the mean time of rhGH therapy of 5.3 and 6.5 years. The OGTT was performed at the mean time, regarding adrenarche start of 3.0 ± 4.7 years in group 1 and 3.4 ± 2.9 years in group 2. The mean time regarding CP start was −0.6 ± 4.1 years in group 1 and 2.0 ± 3.4 years in group 2.

Additional disorders, typical for the PWS patients, such as scoliosis or central hypothyroidism occurred with similar frequency in both groups. However, the patients with PA presented with severe psychological disturbances that required the pharmacological therapy less frequently (2 vs. 6 patients, 18 vs. 43%). This can be partially explained by the younger age of the patients in group 1, the present age 16.2 ± 6.2 vs. 20.8 ± 4.7 years.

## 6. Discussion

One of the commonly described PWS characteristic is hypogonadotrophic hypogonadism and therefore, delayed or incomplete central puberty. Many patients, both females and males, require sex steroids supplementation, what we have also observed in our groups of PWS patients [6,10,24,25]. The PWS boys in the neonatal period are characterized by cryptorchidism, that was present in 9 out of 12 boys in our 2 groups of patients (75%). There are data regarding the possibility of assessment the hypogonadotrophic hypogonadism in the period typical for minipuberty, that seems to be the important part of male genital development [26,27].

However, there is also a hypothesis that the gonadal dysfunction in the PWS patients is heterogeneous [11,12,13,14,15,16]. Apart from the hypothalamic disorder the primary gonadal failure is discussed.

Interestingly, CPP cases have also been reported, as in one of our PWS female patients [17,18]. The timing of pubertal development is primarily genetically driven, mutations of one of the described genes, paternally expressed MKRN3, have been reported to be a cause of CPP [19,20,28]. However, our patient with PA and CPP didn’t present any of the MKRN3 gene mutations.

The frequency of PA in the PWS patients have been assessed as 14–30%, in our study we have documented the similar frequency of PA—22.5%. The origin of this process seems to be undefined. There are data showing that the androgens levels, that are slightly elevated, normalize in adulthood. The rhGH treatment seems to have no effect on the onset of adrenarche or DHEAS levels. The time between the start of rhGH and the onset of adrenarche was similar in the patients with PA and with normal adrenarche in our observation. Adrenarche has been also linked to advanced bone age or obesity previously, which we didn’t prove in our group of patients. Usually, PA in the PWS population is not rapidly progressive and does not lead to CPP. We have described only one girl with PA and CPP [21,22,23].

The PA course in our PWS patients seems to not change the effects and safety of rhGH treatment. We presented the data regarding the improvement in height SDS, GV as well as maintaining the BMI within the normal range during the therapy, regardless of the time of adrenarche. We also didn’t observe the advancement in BA. The metabolic state was comparable in the both groups and there were no more side effects in the AP group. Surprisingly, more psychological disturbances were seen in the group with normal adrenarche. However, the patients in this group were older, which is concordant with the clinical picture of PWS.

There are data suggesting the negative effects of precocious/early adrenarche on future metabolic state, especially in women with a history of PA [29,30]. However, recent studies showed that there is no increased cardiometabolic risk in prepubertal children correlated with higher DHEAS [31]. In our AP group, the girls were predominant, during the follow-up more than half of them presented either impaired glucose tolerance or insulin resistance, only one had minor hypercholesterolaemia and hypertrigliceridaemia. We did not correlate the androgens’ levels and the insulin resistance evaluation results in the PA group as the number of patients was small and not all the children had the full androgen assessment.

In view of the possibility of continuing the rhGH therapy in adult PWS patients it could be worthwhile to assess the metabolic profile according to the history of premature/early adrenarche in childhood.

## 7. Conclusions

The premature adrenarche was present in almost one quarter of our group of PWS patients treated with rhGH. The course of this specific phase of puberty has not been related to the obesity, high androgens’ levels nor advanced bone age as well as the rhGH dose or IGF1 levels. It has also not progressed to central precocious puberty, except in one girl. The timing of central puberty does not seem to be correlated with the time of adrenarche in the PWS patients. Therefore, premature/early adrenarche may be a part of the clinical picture of PWS, apart from hypogonadotrophic hypogonadism.

The safety and the efficacy of the rhGH treatment and the metabolic profile has been similar in the PWS patients with premature and normal adrenarche.

## Figures and Tables

**Table 1:** Group 1. Clinical characteristics at birth and genetic diagnosis. Patient number 10 developed central precocious puberty (CPP).

Patient	Sex	Gestational Age at Birth (Weeks of Pregnancy)	Birth Weight SDS/ Birth Length SDS	Apgar Score (1st Minute of Life)	Age of PWS Diagnosis (Years)	Type of Genetic Diagnosis
1	M	38	−2.35/2.19	6	0.2	abnormal methylation pattern of *SNRPN*
2	F	39	−1.66/1.77	10	0.2	DEL15
3	F	38	−2.73/0.51	9	0.3	DEL15
4	F	38	−2.22/1.57	8	0.4	DEL15
5	M	41	−1.23/1.13	7	0.6	DEL15
6	F	38	−0.88/3.23	10	1.1	DEL15
7	F	40	−3.96/0.31	5	1.1	DEL15
8	M	36	−2.13/2.50	8	1.5	UPD 15
9	F	36	−2.59/0.81	5	2.5	DEL15
10	F	38	−2.70/1.41	10	2.8	abnormal methylation pattern of *SNRPN*
11	F	42	−2.00/1.92	7	3.3	DEL15

M: male, F: female, PWS: Prader-Willi syndrome, *SNRPN*: small nuclear ribonucleoprotein polypeptide N gene, DEL15: deletion of chromosome 15q11-13, UPD 15: uniparental disomy.

**Table 2:** Group 1. The start of the rhGH treatment, onset of adrenarche, bone age and androgens’ levels.

Patient	Age at rhGH Start (Years)	rhGH Dose (IU/kg/Week)	IGF1 SD at rhGH Start	Age of Adrenarche (Years)	BA	DHEAS (ng/mL) (90–720)	Androstenedione (ng/dL) (8–50)	Testosterone (pg/mL) (30–100)
1	2.8	0.38	−0.85	7.5	8.0	1325	ND	248.0
2	1.2	0.60	−1.05	6.2	4.5	612	39.2	ND
3	1.1	0.42	−0.58	5.8	6.8	ND	ND	ND
4	3.1	0.44	−0.79	7.4	7.0	482	51.7	ND
5	5.3	0.80	−1.04	8.4	10.0	1102	48.8	29.0
6	3.4	0.42	−1.33	6.5	6.0	1369	137.0	ND
7	12.3	0.68	−0.61	6.8	8.0	701	40.0	129.0
8	4.3	0.41	−0.30	7.0	6.5	1111	53.9	41.8
9	3.7	0.38	−0.53	6.8	7.0	2068	94.0	ND
10	3.9	0.86	−0.80	6.8	8.8	1742	164.0	72.0
11	8.1	0.49	0.30	6.8	6.0	1772	132.0	95.8

rhGH: recombinant human growth hormone, IGF1: insulin-like growth factor 1, DHEAS: dehydroepiandrosterone-sulphate, BA: bone age, ND: not done.

**Table 3:** Group 1. The metabolic assessment: insulin and glucose in the 0’–120’ of the OGTT, HOMA-IR, lipid profile.

Patient	Age (Years)	Glucose (mg/dL) 0’	Glucose (mg/dL) 120’	Insulin (uIU/mL) 0’	Insulin (uIU/mL) 120’	HOMA-IR	Cholesterol (mg/dL)	LDL (mg/dL)	HDL (mg/dL)	Triglycerides (mg/dL)
1	6.2	91	98	9.2	105	2.1	223	168	39	ND
2	3.4	67	67	6.4	5.8	1.1	181	126	43	62
4	8.9	86	95	7.7	28.9	1.6	173	103	55	77
5	13.6	94	101	9.9	24.4	2.3	170	81	75	68
6	11.0	73	143	10.4	44.0	1.9	152	78	64	62
7	17.5	80	104	11.2	83.3	2.2	208	136	42	152
8	7.6	75	113	31.3	10.2	5.8	116	51	58	36
9	7.5	65	181	8.2	61.9	1.3	193	118	61	70
10	7.3	78	133	15.9	72.1	3.1	143	83	44	83
11	17.7	89	126	16.6	121.0	3.6	184	118	50	83

OGTT: oral glucose tolerance test, HOMA-IR: homeostasis model assessment of insulin resistance, LDL: low density lipoprotein, HDL: high density lipoprotein, ND: not done.

**Table 4:** Comparison group 1 vs. group 2. The rhGH treatment: rhGH dose, height SDS, IGF1 SDS, BA/CA, (the mean value ± standard deviation score, SDS).

Group	Number of Patients	Age at rhGH Start (Years), *p* < 0.05	rhGH Dose (IU/kg/Week; mg/kg/Day)	IGF1 SD at rhGH Start, *p* < 0.05	IGF1 SD GH1	IGF1 SD GH2	BA/CA	Adrenarche Age (Years)	Central Puberty Age (Years)
1	11	4.5 ± 3.2	0.53 ± 0.17; 0.025 ± 0.008	−0.7 ± 0.4	1.7 ± 1.6	1.7 ± 1.7	1.0 ± 0.2	6.9 ± 0.7	12.0 ± 2.2 *
2	14	7.1 ± 2.9	0.50 ± 0.14; 0.024 ± 0.007	−1.1 ± 0.5	1.1 ± 1.5	1.3 ± 1.5	1.0 ± 0.1	10.5 ± 1.3	11.9 ± 1.5 **

RhGH: recombinant human growth hormone, IGF1: insulin-like growth factor 1, GH1: the mean time of rhGH treatment of 1.3 and 1.1 years (groups 1 and 2), GH2: the mean time of rhGH treatment of 5.9 and 6.1 years (groups 1 and 2), BA/CA: bone age/chronological age. * 8 patients, 73% of the group 1, ** 8 patients, 57% of the group 2.

**Table 5:** Comparison group 1 vs. group 2. The rhGH treatment: GV, BMI SDS, HOMA-IR, time of adrenarche and central puberty (the mean value ± standard deviation score, SDS).

Group	Height SD rhGH Start, *p* = 0.15	Height SD GH1	Height SD GH2	GV GH1	GV GH2	BMI SDS rhGH Start, *p* < 0.05	BMI SDS GH1	BMI SDS GH2	HOMA-IR
1	−1.7 ± 1.3	−1.1 ± 1.2	−0.6 ± 1.8	9.0 ± 1.8	6.1 ± 2.0	0.8 ± 1.2	−0.6 ± 1.1	0.6 ± 1.1	2.5 ± 1.4
2	−2.5 ± 1.4	−1.3 ± 1.1	−1.2 ± 0.9	9.6 ± 2.0	5.9 ± 1.0	1.8 ± 1.5	−0.1 ± 1.2	1.1 ± 1.3	3.0 ± 2.0

RhGH: recombinant human growth hormone, GV: growth velocity, GH1: the mean time of rhGH treatment of 1.4 and 1.2 years (groups 1 and 2), GH2: the mean time of rhGH treatment of 6.0 years (groups 1 and 2), BMI: body mass index, HOMA-IR: homeostasis model assessment of insulin resistance.

**Table 6:** Comparison group 1 vs. group 2. The final parameters: total time of rhGH treatment, age, height SDS and BMI SDS at the last visit (the mean value ± standard deviation score, SDS).

Group	rhGH Total Time (Years)	Age at the Last Visit (Years)	Height SD	BMI SD, *p* = 0.12
1	10.2 ± 2.7	14.5 ± 4.0	−1.1 ± 1.5	0.4 ± 1.2
2	8.5 ± 3.1	16.8 ± 2.0	−1.5 ± 1.0	1.2 ± 1.4

RhGH: recombinant human growth hormone, BMI: body mass index.
